# Supplementary material for: Antisense oligonucleotides targeting the miR-29b binding site in the GRN mRNA increase progranulin translation
Source: J Biol Chem. 2023 Nov 18;299(12):105475. doi: 10.1016/j.jbc.2023.105475 (PMC10755782; doi:10.1016/j.jbc.2023.105475)
Supplement: Supporting Figures S1–S10 and Tables S1 and S2 [file mmc1.pdf]

**Antisense oligonucleotides targeting the miR-29b binding site in the *GRN* mRNA increase progranulin translation**

Geetika Aggarwal[1,2,3], Subhashis Banerjee[1,2,3], Spencer A. Jones[1,2,3], Yousri Benchaar[4], Jasmine Bélanger[4], Myriam Sévigny[4], Denise M. Smith[1,2,3], Michael L. Niehoff[1,5], Monica Pavlack[2,3], Ian Mitchelle S. de Vera[2,3], Terri L. Petkau[6], Blair R. Leavitt[6,7,8], Karen Ling[9], Paymaan Jafar-Nejad[9], Frank Rigo[9], John E. Morley[1], Susan A. Farr[1,2,3,5], Paul A. Dutchak[4], Chantelle F. Sephton[4], Andrew D. Nguyen[1,2,3,*]

**Figure S1**

**Figure S2**

**Figure S3**

**Figure S4**

**Figure S5**

**Figure S6**

**Figure S7**

**Figure S8**

**Figure S9**

**Figure S10**

**Table S1**

**Table S2**

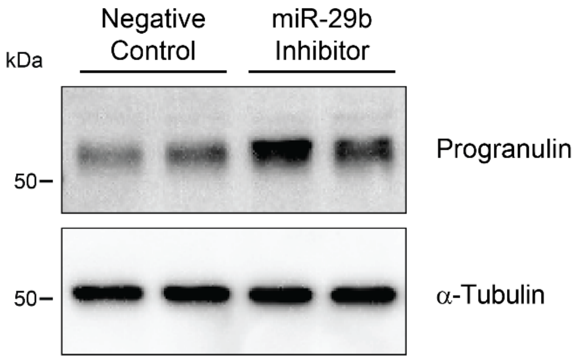


**Figure S1. Broad inhibition of miR-29b increases progranulin protein levels in SH-SY5Y human neuroblastoma cells.** Differentiated SH-SY5Y cells were transfected with 50 nM *mir*Vana miR-29b inhibitor or negative control using Lipofectamine RNAiMAX. After 3 days, progranulin levels in cell lysates were determined by western blot.

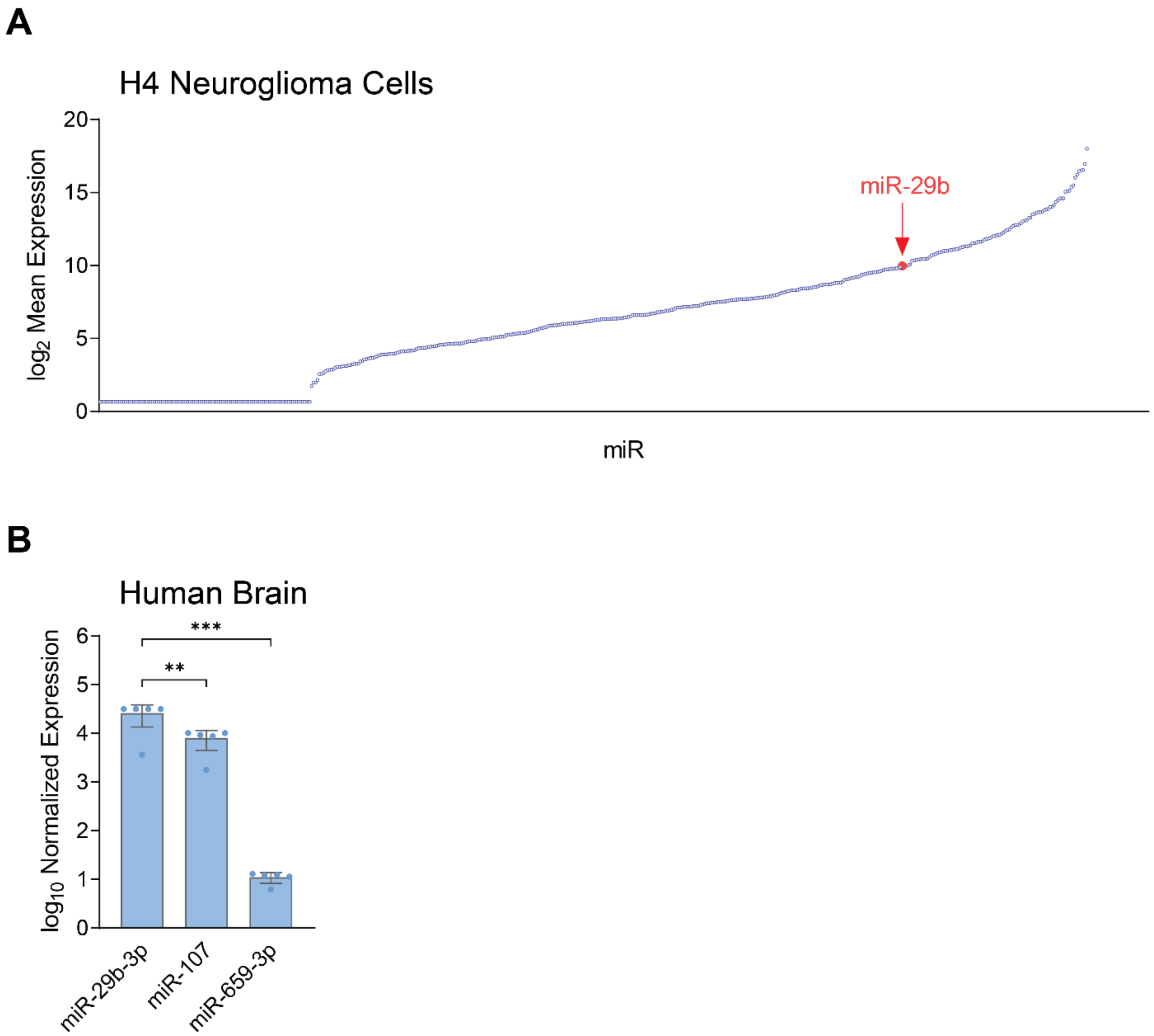


**Figure S2. miR-29b expression levels in cells and tissues.** *A*, miR-29b is moderately expressed in human H4 neuroglioma cells. Ranked profile of miR expression levels, as determined by RNA-seq. miR-29b is indicated in red. *B*, Expression levels of miRs known to regulate progranulin levels in the human brain. Data are presented as means ± SD; individual data points represent technical replicates. ** indicates $p<0.01$ and *** indicates $p<0.001$, as determined by one-way ANOVA with Tukey post hoc test. Data are from the Human miRNA Tissue Atlas (36).

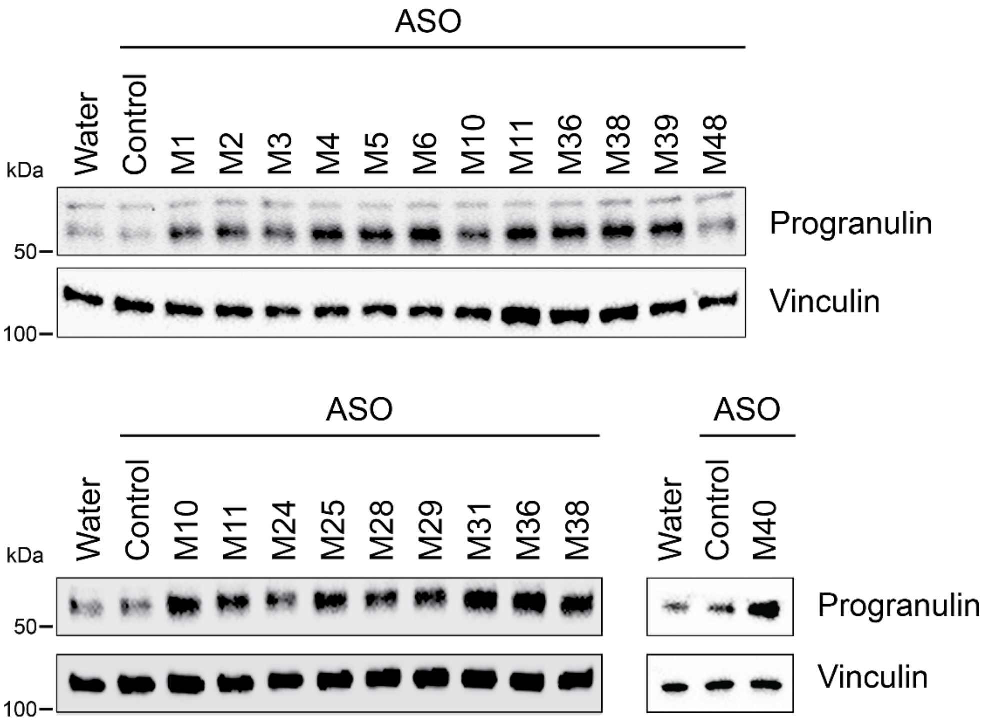


**Figure S3. Western blot validation of candidate ASOs in H4 cells.** H4 cells were treated with 10 μM ASO for 24 h, and progranulin levels in cell lysates were determined by western blot.

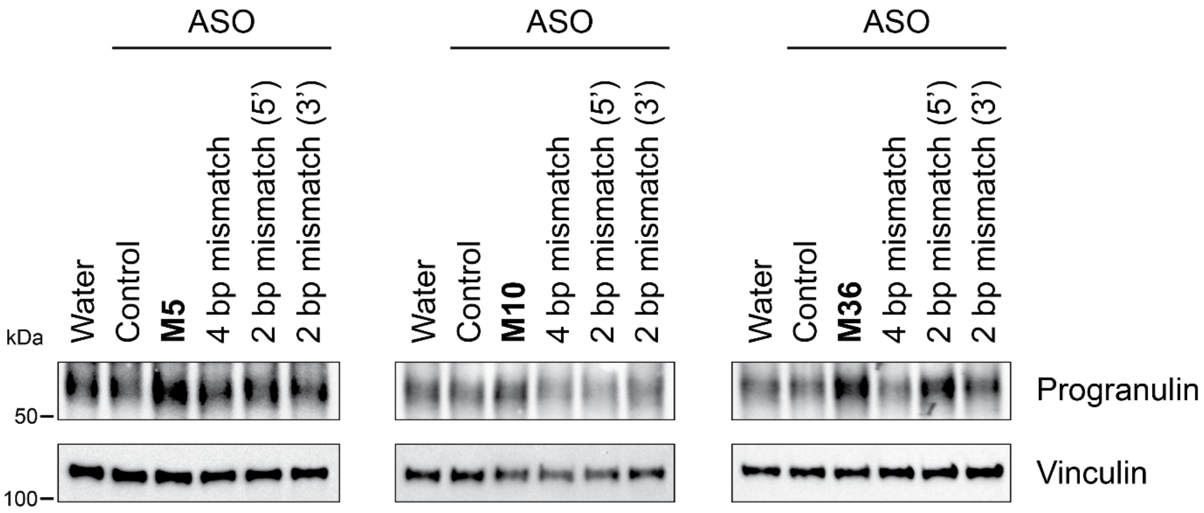


**Figure S4. Most ASOs with 2 bp mismatch lose their activity.** H4 cells were treated with 10 μM ASO for 24 h, and progranulin levels in cell lysates were determined by western blot.

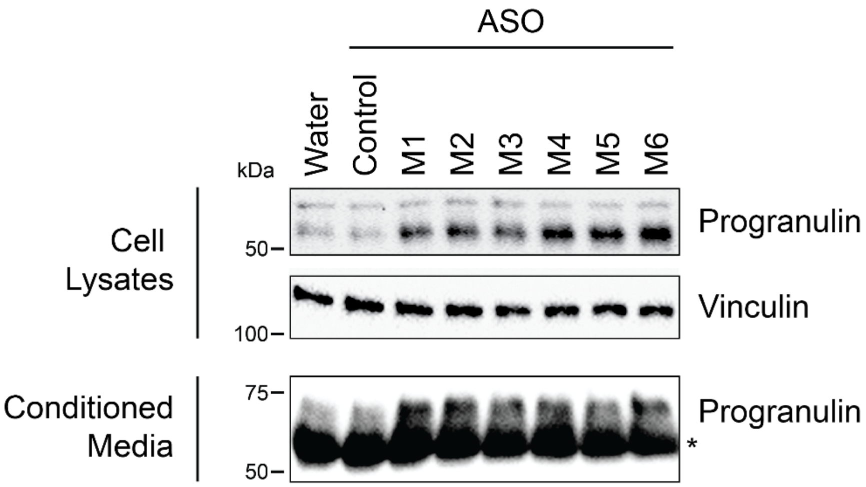


**Figure S5. ASOs increase both intracellular and secreted progranulin levels.** After treating H4 cells with 10 μM ASO for 24 h, progranulin levels in cell lysates and in conditioned media were determined by western blot. * indicates a non-specific band corresponding to albumin. Note, the cell lysate blots are from Figure S3 and are shown here for comparison of the ASO effects on intracellular and secreted progranulin levels.

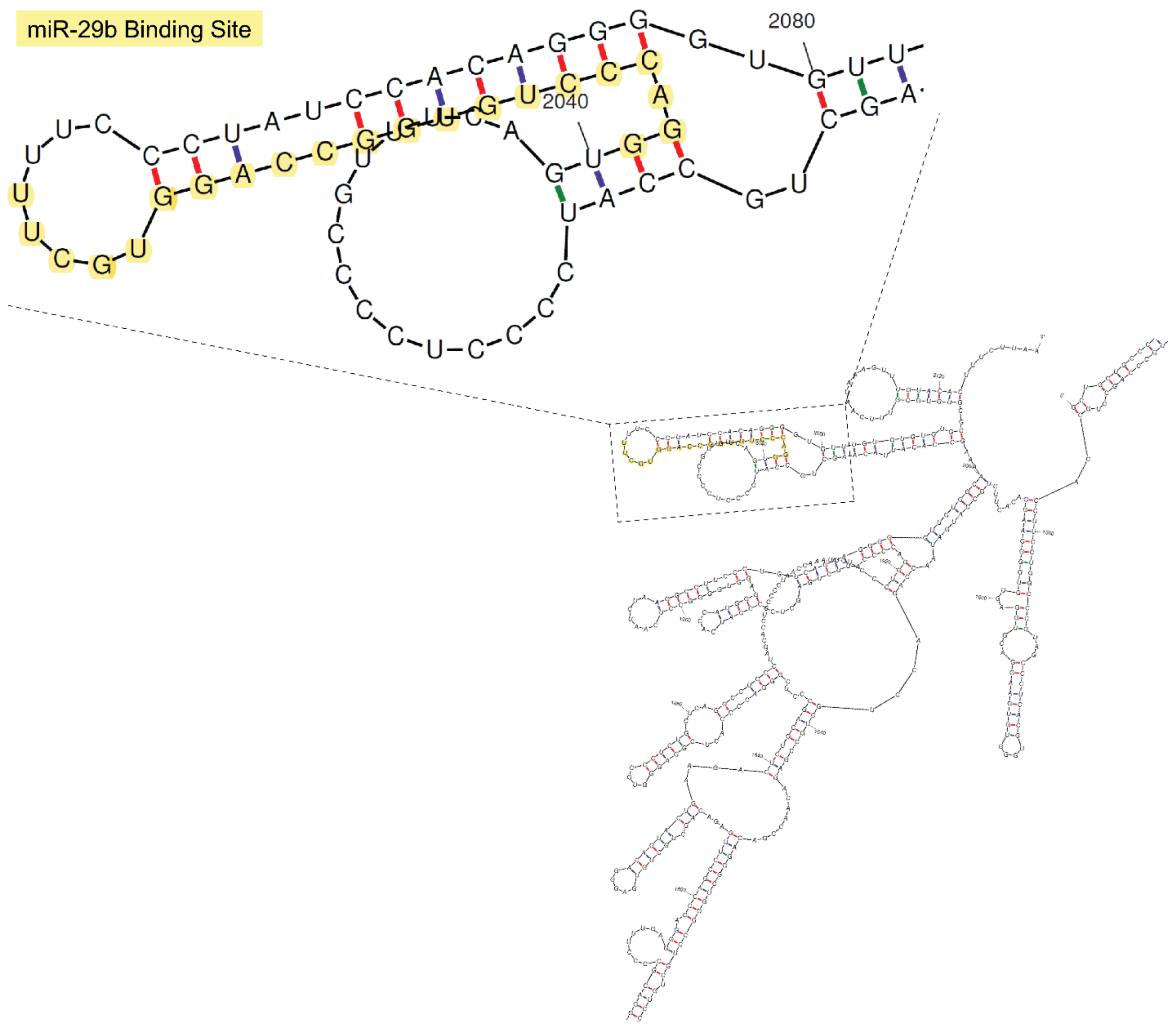


**Figure S6. Predicted secondary structure of the human *GRN* mRNA shows a large accessible loop immediately upstream of the miR-29b binding site.** The mfold program was used to generate the predicted secondary structure. The miR-29b binding site is highlighted in yellow. The nucleotide numbering is based on NCBI reference sequence NM_002087.4, and the 3’ UTR includes nucleotides 1823-2126.

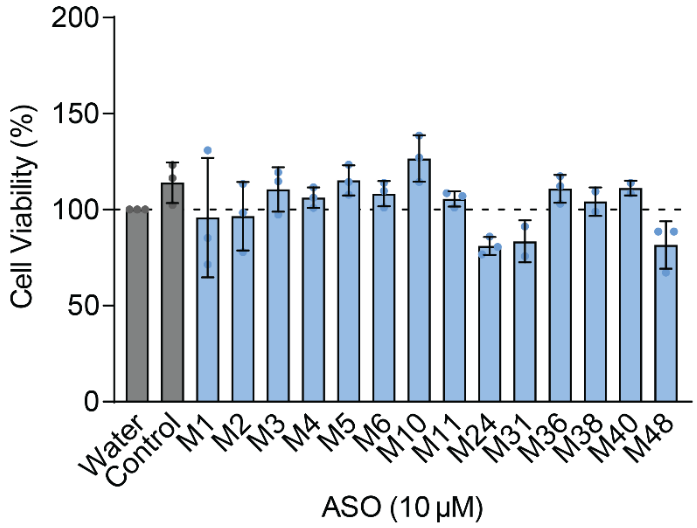


**Figure S7. ASOs are not toxic in cells.** H4 cells were treated with 10 µM ASO for 21 h, and then cell viability was assessed by MTT assay. Data are presented as means ± SD; individual data points represent independent experiments. No statistical differences were found between vehicle- and ASO-treated groups, as determined by one-way ANOVA with Dunnett post hoc test.

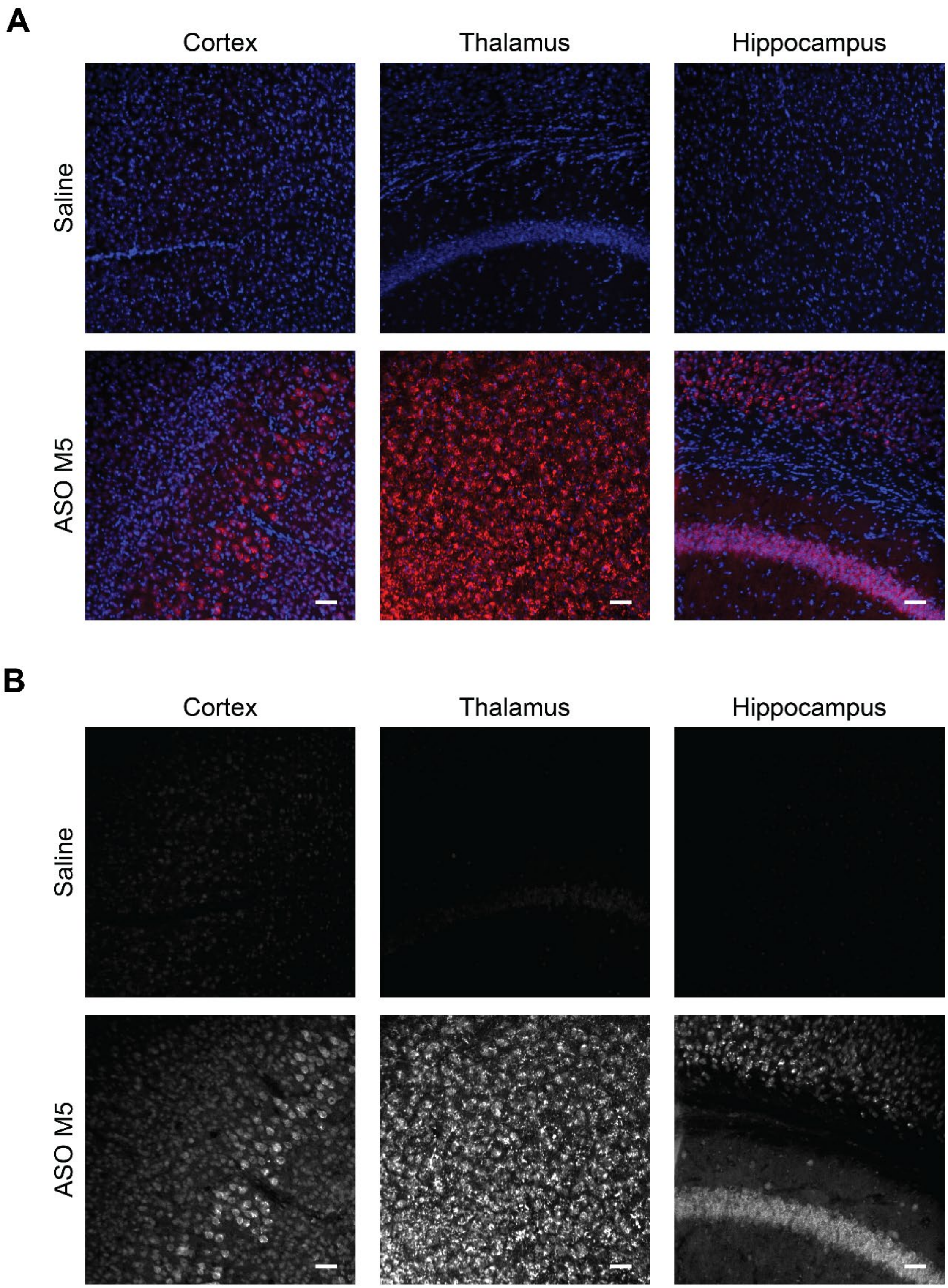


**Figure S8. Confirmation of ASO delivery to the brain.** *A*, At 3 weeks after ICV administration of saline or ASO M5 (500 μg), brains were fixed and sections were stained with an ASO-antibody (red) and counterstained with nuclear stain DAPI (blue). *B*, Gray scale images of ASO staining. Scale bars, 50 μm.

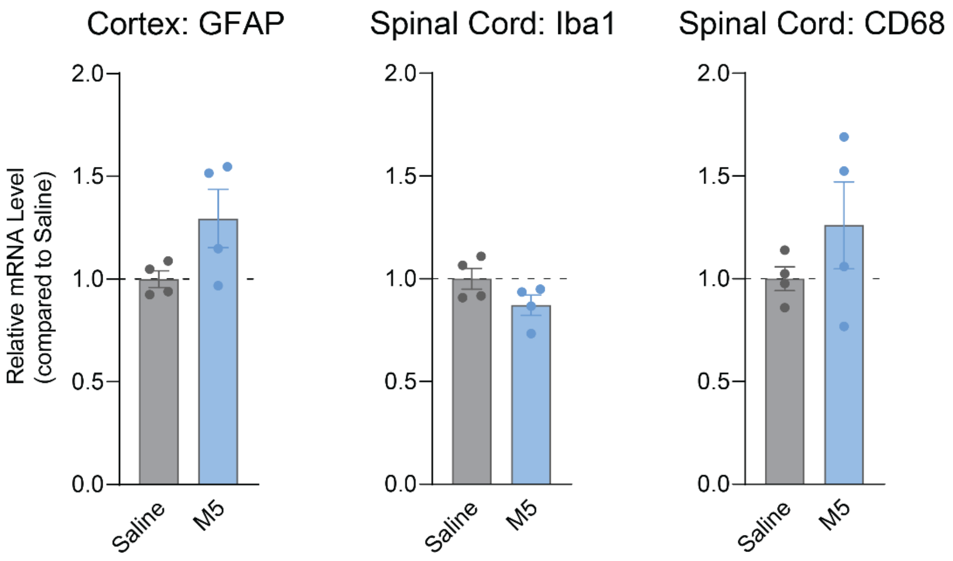


**Figure S9. ASO M5 is tolerated in wild-type mice.** qPCR analysis of inflammatory markers in CNS tissues at 3 weeks after ICV administration of 700 µg ASO or saline vehicle. Data are presented as means ± SEM; data points represent tissues from individual mice. No statistical differences were found between groups, as determined by Mann-Whitney test.

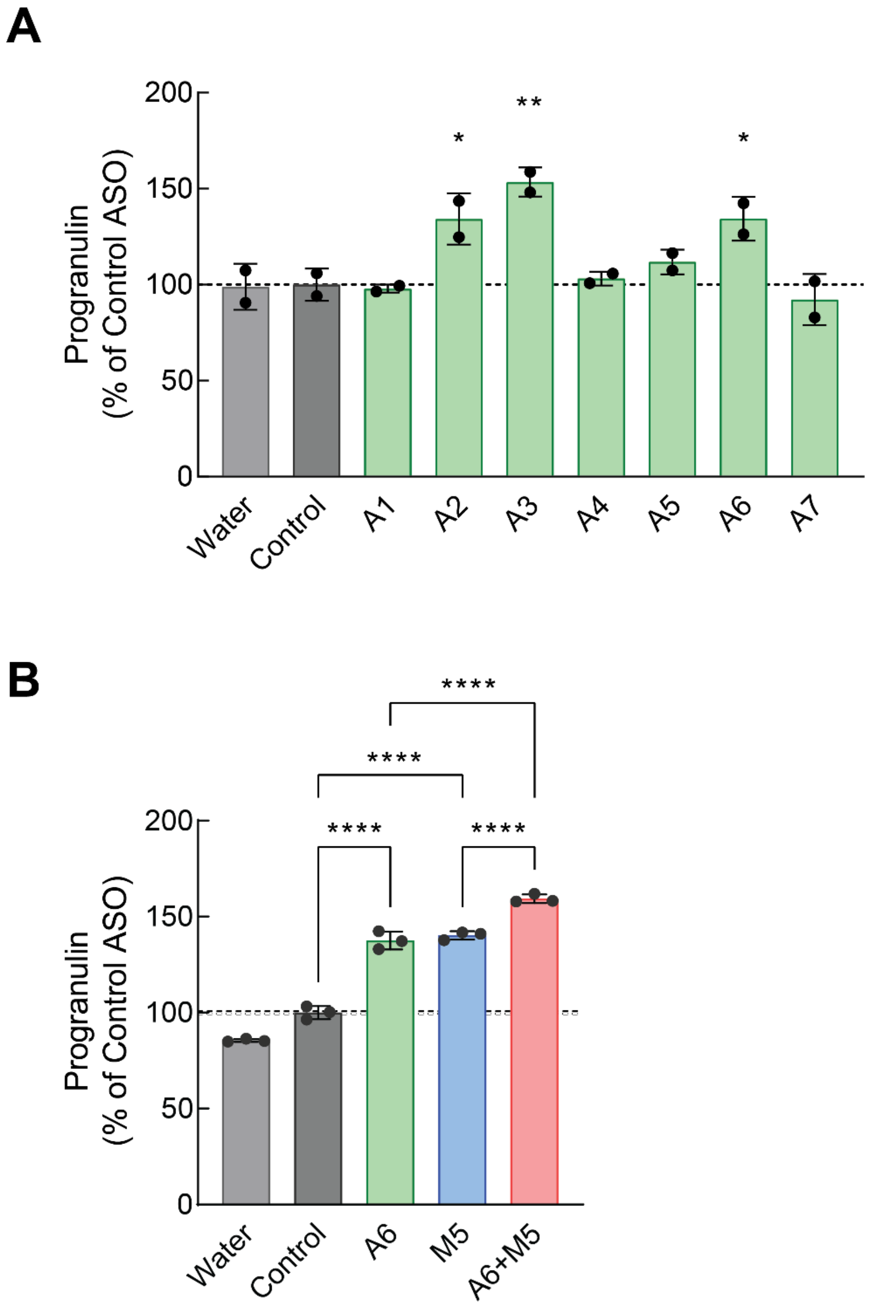


**Figure S10. Several ASOs targeting the miR-659 binding site in the *GRN* 3' UTR increase progranulin protein levels.** *A*, H4 cells were treated with 5 μM ASO for 24 h, and progranulin levels were measured in cell lysates by ELISA. *B*, H4 cells were treated with 80 μM total ASO (40 μM for each ASO plus 40 μM control ASO, or 80 μM control ASO) for 24 h, and progranulin levels were measured in cell lysates by ELISA. Data are presented as means ± SD; individual data points represent biological replicates. * indicates $p<0.05$, ** indicates $p<0.01$, and **** indicates $p<0.0001$, as determined by one-way ANOVA with Dunnett post hoc test.
